# Using Concept Mapping to Develop a Conceptual Framework for Creating Virtual Communities of Practice to Translate Cancer Research into Practice

**DOI:** 10.5888/pcd11.130280

**Published:** 2014-04-24

**Authors:** Cynthia A. Vinson

## Abstract

**Introduction:**

Translating government-funded cancer research into clinical practice can be accomplished via virtual communities of practice that include key players in the process: researchers, health care practitioners, and intermediaries. This study, conducted from November 2012 through January 2013, examined issues that key stakeholders believed should be addressed to create and sustain government-sponsored virtual communities of practice to integrate cancer control research, practice, and policy and demonstrates how concept mapping can be used to present relevant issues.

**Methods:**

Key stakeholders brainstormed statements describing what is needed to create and sustain virtual communities of practice for moving cancer control research into practice. Participants rated them on importance and feasibility, selected most relevant statements, and sorted them into clusters. I used concept mapping to examine the issues identified and multidimensional scaling analyses to create a 2-dimensional conceptual map of the statement clusters.

**Results:**

Participants selected 70 statements and sorted them into 9 major clusters related to creating and sustaining virtual communities of practice: 1) standardization of best practices, 2) external validity, 3) funding and resources, 4) social learning and collaboration, 5) cooperation, 6) partnerships, 7) inclusiveness, 8) social determinants and cultural competency, and 9) preparing the environment. Researchers, health care practitioners, and intermediaries were in relative agreement regarding issues of importance for creating these communities.

**Conclusion:**

Virtual communities of practice can be created to address the needs of researchers, health care practitioners, and intermediaries by using input from these key stakeholders. Increasing linkages between these subgroups can improve the translation of research into practice. Similarities and differences between groups can provide valuable information to assist the government in developing virtual communities of practice.

## Introduction

Communities of practice exist within and across organizations and in our personal and professional lives. They are made up of people who share a common concern, passion, or interest in a topic and choose to actively engage with others to share information and learn from each other. Research on communities of practice has proliferated over the past decade; however, there is limited information on how to create and sustain government-sponsored virtual (Web-based) communities (1–3). Although communities of practice can be launched online, building an online community does not guarantee that people will actively engage in it (4). The objective of this study was to use concept mapping to create a conceptual framework to identify the needs of potential community members and how they would use a federally sponsored virtual community to move cancer research into practice. I solicited input from a target audience of researchers, health care practitioners, and intermediaries (those active in advocacy, philanthropy, funding, federal government, local government, and state government) to understand how to create federally sponsored virtual communities of practice.

Although the goal for all health research, including cancer control, is to improve health outcomes, the percentage of health research findings that are actually put into practice remains small, and transferring research into practice is a lengthy process (5,6). In 2001, the Institute of Medicine highlighted the importance of improving the dissemination of research findings and their implementation in clinical practice. The report, *Crossing the Quality Chasm*, recommends developing new infrastructures to facilitate the translation of research into practice (7). Communities of practice can play a role in enhancing these infrastructures; however, virtual communities of practice are new to the federal government. The National Cancer Institute (NCI) launched Research to Reality in 2011 as one of the first virtual communities of practice at NCI. Evaluations of virtual communities, such as NCI’s evaluation of Research to Reality, are being conducted, but there is limited research on what is necessary to create and sustain federally sponsored virtual communities of practice and how to disseminate and implement these communities. Because the federal government is in the early stages of developing virtual communities, input is needed on what will contribute to their success.

## Methods

I used concept mapping, an integrated mixed-methods approach, to examine the issues key stakeholders believed need to be addressed to create and sustain government-sponsored virtual communities of practice to integrate cancer control research, practice, and policy. Concept mapping is a sequential, mixed-methods planning and evaluation approach that integrates qualitative processes (ie, brainstorming, categorizing ideas, and rating ideas) with multivariate statistical analyses (ie, creating a similarity matrix, multidimensional scaling, and hierarchical cluster analysis) to create visual representations of data (8,9). The research approach used an on-line concept mapping exercise that was conducted in 3 phases. The first phase was the qualitative phase and consists of an online brainstorming project. The second phase was the quantitative phase during which key stakeholders are asked to sort and rate the statements generated in the first phase of the project. The final phase involved having representatives from a subset of the original participant group help interpret the results of the project.

I chose concept mapping for this research because it allows for broad input from a variety of stakeholders across the United States, which is important because the anticipated participants in the community are located throughout the country. In addition, because the focus of the research was on “virtual” communities, it was necessary to conduct research with stakeholders in a virtual environment, that is, via Web-based interaction.

The first phase of the study was brainstorming. From November 21, 2012, through December 2, 2012, members of the Cancer Control P.L.A.N.E.T. (Plan, Link, Act, Network with Evidence-based Tools) website listserv (N = 1,500) were invited to brainstorm ideas on a dedicated website. This listserv was selected because it included a broad range of researchers, practitioners, and intermediaries from across the country. The goal of the brainstorming was to have a mix of researchers, practitioners, and intermediaries come up with from 100 to 250 statements with each statement setting forth an idea that could facilitate transfer of research into practice. Participants were asked to contribute a statement formed by completing the following focus prompt: “*One issue that should be addressed in order to create successful government-sponsored virtual communities of practice designed to move cancer control research into practice is . . ..”* I was unable to determine the number of participants in the brainstorming phase because responses to the focus prompt are anonymous, no log-in is required to participate, and participants could enter multiple responses. During this first phase 193 original statements were generated.

At the conclusion of phase 1, following brainstorming, a small advisory group of experts from NCI who work on virtual communities of practice met to review the brainstormed statements and determine their responsiveness to the focus prompt. During a facilitated discussion in which we used key word groupings for the statements, the group merged similar statements and eliminated statements that were not directly related to the original focus prompt. They reduced the original 193 statements to 70 statements, which were then used for the second phase of the research project. Other concept-mapping projects have used this synthesis-and-reduction process to generate a final statement set that is manageable for sorting and rating (10,11).

The second phase of the study required both sorting the statements into conceptual groups, or clusters, and rating statements on importance and feasibility. The average number of participants involved in sorting and rating statements for Web-based concept-mapping ranges from 28 to 113 (12). Sorting involved the most time and had the lowest participation rate. The original research plan called for sending e-mail invitations in early December 2012 to 50 researchers, practitioners, and intermediaries and asking them to both sort the statements into categories on the basis of their perceived meanings or themes and to rate statements on importance and feasibility on a scale of 1 to 5 (1 = least important or feasible, 2 = somewhat important or feasible, 3 = moderately important or feasible, 4 = important or feasible, and 5 = most important or feasible). Once the initial sorting was complete, e-mail requests were then going to be sent to the Cancer Control P.L.A.N.E.T. listserv asking members to participate in rating statements only. However, the initial response to the sorting and rating request to the 50 researchers, practitioners, and intermediaries was low. To ensure adequate representation and participation, e-mail invitations were sent to the Cancer Control P.L.A.N.E.T. listserv members asking them to participate in both sorting and rating statements for the project. Participants were asked to identify the type of organization they represented: research (academic research or teaching), practice (community-based education, hospital or clinic, managed-care, private group practice, or worksite) or intermediary (advocacy, philanthropy, funding, federal government research or service, local government, or state government). Participants were also asked to identify their level of expertise in working with online communities of practice on the basis of their own perceptions as “no experience,” “some experience,” “average experience,” and “more experience than most.” Table 1 provides a breakdown of participation rates by sorting, rating importance, and rating feasibility. Table 2 describes organizational background of participants and their experience with online communities. Participation rates were equal to or higher than average participation rates reported by Rosas and Kane in 2012 who reported an average completion rate for Web-based sorting of 52.4% and an average completion rate for first Web-based rating of 68.7% (12).

**Table 1:** Participation Rates by Participant Activity, Concept Mapping of Virtual Communities of Practice to Translate Cancer Research into Practice, 2012–2013

Participant Activity	No. of Participants Started	No. of Participants Completed	Percentage Participants Completed
Sorting	78	39	50
Importance rating	66	57	86.4
Feasibility rating	52	43	65.2

**Table 2:** Background and Experience of Participants Completing All Sorting and Rating (N = 60), Concept Mapping of Virtual Communities of Practice to Translate Cancer Research into Practice, 2012–2013

Organizational Background	N (%)
Research	13 (22)
Practice	20 (33)
Intermediary	17 (28)
Did not respond	10 (17)
**Experience working with virtual communities of practice**	
No experience	6 (10)
Some experience	25 (42)
Average experience	13 (22)
More experience than most	6 (10)
Did not respond	10 (17)

I used Concept Systems GlobalMAX software (Concept Systems Inc, Ithaca, New York) to conduct the analyses. The software uses multidimensional scaling and hierarchical cluster analysis to interpret sorting and rating data and create maps and graphs (9,13). Several different cluster maps were generated, ranging from a 6-cluster solution to a 13-cluster solution. For the final phase of the project, I shared the statements for the different cluster maps during an interpretation meeting with members of the Federal Virtual Communities of Practice workgroup. This workgroup included the leaders of the Public Health Connect community of the Centers for Disease Control and Prevention, the National Cancer Institute’s (NCI’s) Director of the Office of Partnerships and Disseminations Initiatives, managers of NCI’s Research to Reality community, and a community manager for the US Department of Education. After sharing the different cluster map solutions with the workgroup members, I used their input to select a cluster map that was the best fit for depicting the respondent data. I also analyzed the clustered statements from the 3 groups (researchers, practitioners, and intermediaries) by creating pattern-match diagrams. The pattern match describes the average importance ratings for each cluster by each group relative to other clusters. Clusters rated higher in relative importance are located at the top of the scale. However, placement at the bottom of the scale does not mean a cluster is not important to a group; it means the cluster is less important relative to other clusters. The results are best interpreted as qualitative data from 3 focus groups rather than viewed as survey data from individual participants.

In cluster mapping, the position of the individual clusters in relationship to each other is significant as is the placement within the cluster of the number for each statement included in each cluster. The Appendix lists the statements assigned to each cluster and the number associated with the individual statement on the map.

**Figure 1:**
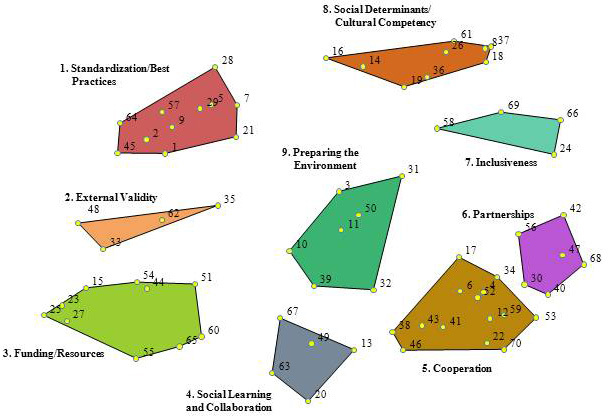
Point cluster map depicting a conceptual framework for creating federally sponsored virtual communities of practices for moving cancer control research into practice. Each number within a cluster indicates a specific statement or issue and how it was sorted in phase 2 of the study (see Appendix for the list of statements associated with each cluster). The position of a statement number within the cluster is significant: it depicts that statement’s relationship to other statements in the figure.

Multidimensional scaling translated the sorting from all participants in the project and placed each concept as a separate point on a 2-dimensional map (Figure 1). The location of each statement matters. Statements that are located closer to each other are more likely to have been sorted together by participants (for example, statement 35 is more likely to have been sorted together with statement 62 in the external validity cluster than with statement 68 in the partnership cluster). A concept map has no X or Y axis. However, the statement location remains fixed in relation to all other statements. Hierarchical cluster analysis shows options for drawing boundaries around statements to create clusters.

In analyzing the results of this study, it is important to address the issue of internal validity. Internal validity is primarily concerned with the ability to draw conclusions about cause and effect of a treatment or intervention. Since this research was not looking at a cause-and-effect relationship, a different focus on internal validity is necessary. For concept mapping projects, internal representational validity looks at the degree of fit of the final concept map to how individuals originally organized and rated statements (13). Internal representational validity was measured by calculating the stress index. According to Kruskal and Wish, the lower the stress value, the better the fit of the multidimensional scaling (MDS), and they recommend a stress value of 0.10 or lower as the standard for MDS (9). However, Kane and Trochim found that for concept mapping projects, this measure is too low and that the average stress value should be 0.285 with a standard deviation of 0.04 (13). The final stress statistic for this study is 0.28.

## Results

Members of the Federal Virtual Communities of Practice workgroup reviewed the different cluster maps. They agreed that individual statements grouped in the 9-cluster map created the best conceptual framework for this study (Figure 1). The complete statement list with corresponding numbers is in the Appendix.

**1. Standardization and best practices.** Statements in this cluster focus on developing guidelines and standards for dissemination and implementation of research results and the need for agreed-upon terms and methods for dissemination and implementation. Specific statements speak to the need for developing measures that will support dissemination and implementation as well the ability to compare data across systems (eg, states, hospitals).

**2. External validity**. Statements in cluster 2 focus on issues related to translation or transferability. This cluster acts as the bridge between the standardization and best practices cluster and the funding and resources cluster and focuses on the need for real-world experience that can be used to justify the use of a research-tested program in clinical practice. Whereas the standardization and best practices cluster speaks about guidelines for dissemination and implementation and the funding and resources cluster focuses on tools and resources for dissemination and implementation, the external validity cluster asks for case studies.

**3. Funding and resources.** Statements in this cluster focus on grant funding mechanisms and creation of resources to support the translation of research into practice. An overall theme of the statements in cluster 3 is creating funding mechanisms that allow for community involvement as well as involvement of multidisciplinary teams. A secondary theme is the need to create or organize tools, education materials, and other resources designed to assist with the translation of research into practice.

**4. Social learning and collaboration.** Cluster 4 is located in the middle at the bottom of the figure. It is bordered by cluster 3, funding and resources; cluster 5, cooperation; and cluster 9, preparing the environment. The positioning of these 3 clusters illustrates the relationships among research, practice, and collaboration. The theme for this cluster is providing space and opportunities to permit shared learning. For shared learning to occur, participants in the community of practice must have the ability and desire to collaborate. Furthermore, participants need to be able to share information about their work and need their organizations’ support to allow this sharing to happen in a virtual setting. This cluster speaks not only to the need for sharing among public health practitioners, but also to the need for government and other funding officials to participate in the virtual communities. Trust is an overarching issue when considering willingness to share and collaborate.

**5. Cooperation.** The cooperation cluster is on the lower right side of the figure, the side diagonally opposite the standardization and best practices cluster. The cooperation cluster focuses on engaging broad and diverse expertise and organizations in government-sponsored virtual communities of practice. Some statements in cluster 5 seem directly related to the social learning and collaboration cluster (eg, “participant willingness to share work in progress”). However, the majority of statements in this cluster focus on having broad representation for addressing the issues of translating cancer control research into practice.

**6. Partnerships.** The partnership cluster is on the right side of the figure above the cooperation cluster and is closely related to that cluster. Some of the proposed cluster labels were the same for both clusters. This cluster is differentiated from the cooperation cluster by focusing on inclusion of partners at the beginning of research and the beginning of the creation of the virtual community of practice. Several statements specify the types of participants that should be included such as health care providers, office managers, community organizations, health departments, federally qualified health centers, survivors, and family members. Statements in this cluster have a very different focus than the standardization and best practices cluster on the opposite side of the map.

**7. Inclusiveness.** The inclusiveness cluster falls between the social determinants and partnerships clusters because its focus is on making sure a variety of populations, including patients, tribal nations, and health care practices, are represented in the communities.

**8. Social determinants and cultural competency.** This cluster is located at the top of the map between the standardization and best practices and inclusiveness clusters. The social determinants and cultural competency cluster addresses the need to include a focus in the virtual community on issues related to health disparities, social determinants of health, and cultural competency, specifically, how these issues affect an person’s lifetime risk of cancer and also how research is translated into practice in disparate populations.

**9. Preparing the environment.** This cluster is located in the center of the map, indicating by its position that its statements can be related to all the surrounding clusters. Statements in this cluster focus on preparation for moving research into practice.

**Figure 2:**
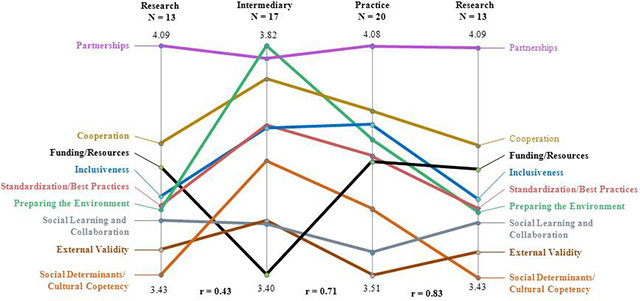
Pattern match of areas of importance for creating government-sponsored virtual communities of practice, by group.

The results from the concept mapping study provide insight into similarities and differences among researchers, practitioners, and intermediaries. Sixty participants completed the sorting and all the ratings, but only 50 answered questions concerning their organizational backgrounds (Table 2). The concept maps generated for the 50 people who responded to the organizational background question showed that researchers, practitioners, and intermediaries were in relative agreement regarding issues of importance in creating a virtual community of practice designed to move cancer control research into clinical practice (Figure 2). On the basis of the pattern match, the 3 groups (researchers, practitioners, and intermediaries) had a correlation regarding the relative importance of creating communities ranging from r = 0.43 to r = 0.83. Participants also helped to organize issues that the federal government should address to develop effective and sustainable virtual communities of practice. However, actively engaging each group may require focusing on different priority areas.

**Table:** 

Figure 2 is a pattern match showing the correlation between the average importance rating for each cluster for each of the 3 subgroups and how each group conceptualizes the issues related to the creation of successful virtual communities of practice. It also shows where there are areas of similarities or differences between groups. The first rating in the figure shows how researchers rated each cluster of issues on an importance range of 3.43.to 4.09. Partnerships were rated highest in importance (4.09) followed in descending order by cooperation, funding and resources, inclusiveness, standardization and best practices, preparing the environment, social learning and collaboration, and external validity. Social determinants and cultural competency was rated the lowest on importance (3.43) by researchers.
The second rating in the figure shows how 17 intermediaries rated the importance of domains of issues with ratings ranging from 3.40 to 3.82. Intermediaries rated preparing the environment highest on importance (3.82) followed in descending order by partnerships, cooperation, standardization and best practices, inclusiveness, social determinants and cultural competency, external validity, and social learning and collaboration. Intermediaries rated funding and resources lowest in importance (3.40). The correlation between research and intermediary ratings was 0.43.
Twenty public health practitioners rated clusters of issues in a range from 3.51 to 4.08. Practitioners rated partnership highest on importance (4.08) followed in descending order by cooperation, inclusiveness, preparing the environment, standardization and best practices, funding and resources, social determinants and cultural competency, social learning and collaboration. Practitioners rated external validity lowest on importance (3.51). The correlation between practitioner and researcher ratings was 0.83, and the correlation between practitioners and intermediaries was 0.71.

The ratings from the pattern match provide insight into how researchers, practitioners, and intermediaries view these issues, which can be helpful when trying to engage these groups to participate and contribute to the virtual communities. Similarities and differences exist among the 3 groups (researchers, practitioners, and intermediaries) around the importance of creating virtual communities of practice designed to move cancer control research into practice. There is a strong correlation between how intermediaries and practitioners (r = 0.71) and how practitioners and researchers (r = 0.83) rated importance of statements included in clusters. This indicates that the 3 groups are in general agreement about what issues need to be addressed during the creation of the virtual community. Researchers and practitioners rated partnership and cooperation as the most important issues that need to be addressed when creating virtual communities of practice. However, intermediaries rated preparing the environment as the most important issue that should be addressed, followed by partnerships. Researchers and practitioners rated issues related to funding and resources similarly toward the middle on importance while intermediaries rated funding and resources as the lowest in importance. Issues related to social determinants and cultural competency were the least important for researchers, and issues related to external validity were the least important for practitioners.

## Discussion

Researchers derive the greatest benefit from the types of partnerships that occur in a virtual community of practice. The ability to partner and learn from other researchers and having the opportunity to develop partnerships with health care practitioners and intermediaries may be key to engaging researchers in a virtual community. Health care practitioners are more likely to be engaged if the virtual community is patient- or client-centered. They will also be more involved if guidelines and resources for translating research into practice are available in an easy-to-use format at little or no cost. Because intermediaries are the people who link researchers and practitioners, promoting the virtual communities as a mechanism for collaboration and partnership may increase intermediaries’ participation.

Trust is a recurring theme throughout the literature on virtual communities of practice and is reflected in my concept mapping study. The literature shows that for individuals to engage actively in a community they must develop trust with other members of the community (14–16). Trust is implied in statements within the cooperation, partnerships, inclusiveness, and social learning and collaboration clusters. People will not share information about their work if they do not trust that members of the community will respect their work. In a virtual community of practice, developing trust may be a challenge because there is limited or no face-to-face interaction. This study did not address specific methods for increasing trust or how that trust could be measured. Exploring issues of trust in a federally sponsored virtual community of practice is a potential area for future research.

The findings from this concept-mapping study not only provide guidance for federally sponsored virtual communities of practice focused on translating cancer control research into practice. Many of the recommendations are relevant for other federal agencies that may be considering to the development such communities on other topics. Other government and nongovernment agencies that are involved in developing virtual communities of practice to translate science into practice can benefit from understanding the different perspectives of researchers, practitioners, and intermediaries when developing strategies to engage these key groups.

## Appendix. Using Concept Mapping to Develop a Conceptual Framework for Creating Virtual Communities of Practice to Translate Cancer Research into Practice: Statements for Each Cluster

This file is available for download as a Microsoft Word document.

## References

[1] Wenger E , Trayner B , de Latt M . Promoting and assessing value creation in communities and networks: a conceptual framework. Open Universiteit, Ruud de Moor Centrum; 2011;18:1-56. http://wenger-trayner.com/documents/Wenger_Trayner_DeLaat_Value_creation.pdf. Accessed 19 February 2014.

[2] Li LC , Grimshaw JM , Nielsen CP , Judd M , Coyte PC , Graham ID . Evolution of Wenger's concept of community of practice. Implement Sci 2009;4:11. 10.1186/1748-5908-4-11 19250556 PMC2654669

[3] Millington R . Buzzing Communities: How to build bigger, better, and more active online communities. FeverBee; 2012. http://www.feverbee.com/2012/11/buzzing-communities-.html. Accessed 19 February 2014.

[4] Brazelton J , Gorry GA . Creating a knowledge-sharing community: if you build it, will they come? Commun ACM 2003;46(2):23–5. 10.1145/606272.606290

[5] Balas EA , Boren SA . Managing clinical knowledge for health care improvement. Section 1: health and clinical management. In: Bemmel J, McCray AT, editors. Yearbook of medical informatics: patient centered systems. Stuttgart (DE): Schattauer Verlagsgesellschaft; 2000. p. 65–70. 27699347

[6] Glasgow RE , Vinson C , Chambers D , Khoury MJ , Kaplan RM , Hunter C . National Institutes of Health approaches to dissemination and implementation science: current and future directions. Am J Public Health 2012;102(7):1274–81. 10.2105/AJPH.2012.300755 22594758 PMC3478005

[7] Institute of Medicine. Crossing the quality chasm: a new health system for the 21st century, 2003. Washington (DC): Institute of Medicine; 2001.

[8] Trochim WMK . An introduction to concept mapping for planning and evaluation. Eval Program Plann 1989;12(1):1–16. 10.1016/0149-7189(89)90016-5

[9] Kruskal JB , Wish M . Multidimensional Scaling. Beverly Hills, CA: Sage; 1978.

[10] Anderson LA , Day KL , Vandenberg AE . Using a concept map as a tool for strategic planning: The Healthy Brain Initiative. Preventing Chronic Disease 2011;8(5):A116. PMC3181190 21843420

[11] Graham AL , Kerner JK , Quinlan KM , Vinson CA , Best A . Translating cancer control research into primary care practice: a conceptual framework. Am J Lifestyle Med 2008;2:241–249. 10.1177/1559827608314146

[12] Rosas SR , Kane M . Quality and rigor of the concept mapping methodology: a pooled study analysis. Eval Program Plann 2012;5;35(2):236-245. 10.1016/j.evalprogplan.2011.10.003 22221889

[13] Kane M , Trochim WMK . Concept mapping for planning and evaluation. Thousand Oaks (CA): Sage Publications; 2007.

[14] Wenger E . Communities of practice: learning, meaning, and identity. Cambridge (UK), New York (NY): Cambridge University Press; 1998.

[15] Lave J , Wenger E . Situated learning: legitimate peripheral participation. Cambridge (UK), New York (NY): Cambridge University Press; 1991.

[16] Kim A . Community building on the Web. Berkeley (CA): Peachpit Press; 2000.

