# Supplementary material for: Using Concept Mapping to Develop a Conceptual Framework for Creating Virtual Communities of Practice to Translate Cancer Research into Practice
Source: Prev Chronic Dis. 2014 Apr 24;11:E68. doi: 10.5888/pcd11.130280 (PMC4008948; doi:10.5888/pcd11.130280)
Supplement: Supplementary file 1 [file 13_0280_01.doc]

Appendix. Using Concept Mapping to Develop a Conceptual Framework for Creating Virtual Communities of Practice to Translate Cancer Research into Practice: Statements for Each Cluster

| **Cluster Name** | **Statement No.** |
| --- | --- |
| ***1. Standardization/Best Practices*** | |
| To establish priority areas for development/delivery/dissemination. | 1 |
| Development of consistent language/terms, methods, etc. | 2 |
| Best practice guidelines that are realistic for community cancer programs. | 5 |
| Having guidance about which research evidence is most relevant to my organization. | 7 |
| The value of clinical trials, which are often negatively stigmatized. | 9 |
| Research that better assesses the factors influencing research translation. | 21 |
| Standardized, professional marketing tools and resources that consider language and comprehension levels. | 28 |
| National benchmarks that allow comparison of data between states and individual reporting hospitals. | 29 |
| To focus on implementation science. | 45 |
| Support the addition of measures and questions to surveillance systems to help promote research translation. | 57 |
| Identifying standardized measures/metrics to quantify “successful” implementation of research into practice in a crowd sourced way. | 64 |
| ***2. External Validity*** | |
| Real world case studies of actual (varying) budgets in which communities can virtually plan research programs and how they will work in their own community. | 33 |
| Planning for community implementation to be a time of active improvement of the VCoP based on rapid-cycle user feedback. | 35 |
| Sustainability business cases to allow implementation of evidence based practice. | 48 |
| That research needs to be sell-able at my organization. I need a business case. | 62 |
| ***3. Funding/Resources*** | |
| Creation of mini-grants for CoPs that select and conduct demonstration or implementation projects regarding moving cancer control research into communities or clinical practices. | 15 |
| Lower the barriers and red tape in funded projects to allow more freedom in communities to decide where funds should be utilized. | 23 |
| Priority funding that can be set aside for participants (particularly practitioners) related to the topic of interest. | 25 |
| Reduce funding barriers to allow for more flexibility of inter disciplinary teams. | 27 |
| More transparent community driven grant processes, not grant driven limited data and disparities. | 44 |
| Develop a repository for practice guidance and allow users to comment on how useful that material was or how they adapted it (e.g., allrecipes.com). | 51 |
| Centralize and organize all tools and health education materials developed through research grants. | 54 |
| Make the utilization/participation in a government-sponsored virtual community of practice as a requirement for government funded grants. | 55 |
| Ensure sharing of researcher information by making it a requirement of funding. | 60 |
| Support learning for workers with limited funds for training who are interested in translating research into practice. | 65 |
| ***4. Social Learning & Collaboration*** | |
| Provide opportunities for members to showcase their work and programs, so that others can contact them to collaborate or learn from each other. | 13 |
| Encourage state governments to be responsive in a virtual setting. With limited access to individual program or even section/division social media formats, managing a meaningful virtual community of practice could be challenging for some states. | 20 |
| Consolidate prevention campaigns for breast, colon and prostate cancers as one prevention effort. | 49 |
| Have program officers and other funding officials actively involved in discussion groups to clarify government policies and positions. | 63 |
| Create a national mentorship program for practitioners beyond clinical practice. | 67 |
| ***5. Cooperation*** | |
| Work across agencies and disciplines to create useful cancer surveillance tools for cancer incidence and mortality at the finest granularity possible. Partners could include CDC, GIS experts, cancer registries, & cancer advocacy organizations. | 4 |
| Encourage a team science or transdisciplinary approach. | 6 |
| Provide cross-over information from other specialties throughout the chronic disease repertoire. | 12 |
| Bridge clinical practices with community-based approaches. | 17 |
| Need to bring governmental public health agencies together with private organizations to test ways to implement evidence-based practices. | 22 |
| Integration with existing virtual communities (many providers are involved in multiple “discussion boards/networks/listservs/blogs/etc”) considering how to make this meaningfully interconnected with options that already exist. | 34 |
| Assuring the linkage between universities that do animal research for cancer with hospitals and medical providers to reduce redundancy in some equipment and laboratory capabilities as well as improving learning from each other. | 38 |
| Participant willingness to share work in progress. | 41 |
| How to most efficiently and effectively identify topics of mutual interest to the government as well as the CoP participants. | 43 |
| Creation of a norm of open (as opposed to proprietary) sharing among practitioners employed by different and even rival organizations; the normative appeal should be to the field or discipline (“state of the art of the practice”) rather than organizational. | 46 |
| Devote time and effort into developing partnerships between the research and practice communities. Both communities are susceptible to acting in silos. | 52 |
| Engage all community organizations (not just gov't) implementing cancer prevention and control efforts. | 53 |
| Create and maintain collaborative relationships between academic researchers and community leaders outside government to foster research to reality transitions. | 59 |
| Public-private partnerships with health care institutions, especially bill-pay institutions, to better assess the efficiency and effectiveness in the delivery of cancer-related services to those populations being diagnosed with cancer. | 70 |
| ***6. Partnerships*** | |
| Bringing providers, office managers, mid-level professionals, health departments. federally qualified health centers, etc. into the discussion to test the research applicability in the real world setting they operate in. | 30 |
| Identification of key stakeholders at all levels of implementation. | 40 |
| Inclusion of survivors and family members or caretakers. | 42 |
| Community organizations/members should be engaged from the beginning of building the virtual community to make is successful. | 47 |
| Better engage/get buy in from community organizations from the beginning of the research to help move the results into practice. | 56 |
| Engage all community organizations on cancer prevention. Starting with educating children at a early age the importance of taking care of our health. | 68 |
| ***7. Inclusiveness*** | |
| Make sure patients perspectives are central to the focus of the community of practice. | 24 |
| Incorporation of patient-reported outcomes to identify areas of unmet need. | 58 |
| Successfully adopt a plan to work with Tribal Nations engaging community support but also working with the Tribal Institutional Review Boards to ensure accountability for all data. | 66 |
| An understanding of both the practice's (clinic, public health, worksite, policy, etc) specific needs/culture and the aspects of the research that “fit” that culture. | 69 |
| ***8. Social Determinants/Cultural Competency*** | |
| Represent and be sensitive to health issues in disparate populations (e.g. rural, Appalachian, African American). | 8 |
| The value of alternative treatments other than western practices. | 14 |
| Establishing standardized, cross cultural training and certification for cancer educators across the cancer control continuum. | 16 |
| How characteristics of “place” and geography, combined with related upstream factors which may be cultural, racial, political, economic, etc., may be determining much of our lifetime cancer risk. | 18 |
| Inclusion of social determinants of health/socio-ecological model in the framework to put research into practice - that environment plays a major role in making healthy decisions. | 19 |
| Continuing to study health equity and find novel ways to approach changing health behaviors for underserved communities. | 26 |
| Geospatial references to high risk target populations that allow virtual communities to implement more specific, community-based action plans within their immediate environments (one size does not fit all). | 36 |
| How culture is a large part of the make-up of a community. What works in one community may or may not work in another. | 37 |
| That race, ethnicity, and culture need to define the subgroups so one can easily pick and choose what is of interest to their work or project needs. | 61 |
| ***9. Preparing the Environment*** | |
| Research into how organizations, teams, and individuals affect the cancer control and the cancer care delivery process. | 3 |
| More practitioners doing research. | 10 |
| Real world practice discussions to assure that we don't develop solutions looking for problems. | 11 |
| Sharing evidence-based campaigns among interdisciplinary professionals and toward various ethnicities. | 31 |
| Promote and provide pathways for cancer control research collaboration therefore increasing efficiency and results while reducing redundancy and costs. | 32 |
| Sharing research findings in a way that describes and supports effective policy/environmental change activities at the local, state, and federal level by public health and health practitioners. | 39 |
| Access to and sharing of population-based statistics on cancer screening, diagnosis, and treatment that can identify high-risk target populations at the community/county/neighborhood level (i.e. claims data for screening and Registry data for late stage) at one website. | 50 |
